# How do people with mood and anxiety disorders perceive and interpret the Drinking Motives Questionnaire? A think-aloud study in a clinical setting

**DOI:** 10.1186/s13722-018-0109-1

**Published:** 2018-03-14

**Authors:** Christina Nehlin, Margareta Wennberg, Caisa Öster

**Affiliations:** 1 Department of Neuroscience, Psychiatry, Uppsala University, UAS ing 10, 75185 Uppsala, Sweden; 2 Division of Psychiatry, Uppsala University Hospital, UAS ing 10, 75185 Uppsala, Sweden

**Keywords:** Questionnaires, Drinking motives, DMQ-R, Think-aloud method, Psychiatric care

## Abstract

**Background:**

Research has identified drinking motives as the final common pathway to alcohol use, and associations between specific drinking motives and drinking patterns have consistently been demonstrated. Data on drinking motives can be used for research, in the planning of prevention strategies and for treatment purposes. The Drinking Motives Questionnaire-Revised (DMQ-R) has become the most used measure of drinking motives. So far, the questionnaire has not been investigated with qualitative methods. The aim of this study was to investigate acceptability, accuracy and usability of the DMQ-R among persons receiving outpatient psychiatric care by studying how responders perceive and interpret the questionnaire.

**Method:**

A cognitive interviewing technique, the think-aloud method, was used to collect data from 16 non-alcohol dependent patients seeking outpatient psychiatric care (12 women, 4 men). To analyse data, Qualitative Content Analysis was applied in which themes were formed from data only and not from predetermined areas of interest.

**Results:**

Overall, acceptability of the DMQ-R was high although answers were sometimes given with low accuracy. Responders pointed out that they perceived the questionnaire as non-confrontational and exhaustive. Further, the DMQ-R seemed to launch processes of self-reflection.

**Conclusions:**

Taken together, the results suggest a support for the use of DMQ-R also in the group of psychiatric outpatients. Still, when interpreting the DMQ-R, a certain insecurity of the exactness of answers should be considered. The graphic design should be particularly clear in this group of patients.

**Electronic supplementary material:**

The online version of this article (10.1186/s13722-018-0109-1) contains supplementary material, which is available to authorized users.

## Background

Drinking motives have drawn increasing interest because of their role in the development of alcohol-related problems. Research has identified drinking motives as the final common pathway to alcohol use [1, 2]. Associations between specific drinking motives and drinking patterns have consistently been demonstrated (e.g. [3–5]). For example, emotionally oriented motives are more often associated to high levels of drinking and to negative consequences [6]. Data on drinking motives may be useful for research purposes, when planning of prevention strategies and for tailoring of treatment.

The Drinking Motives Questionnaire-Revised (DMQ-R) [1] is a self-report questionnaire to assess the relative frequency of four separate motives for drinking. The questionnaire was developed in 1994 for use in college student populations. It has become the most used measure of drinking motives; the original study by Cooper [1] has been cited over 1000 times (Scopus, November 2017). Most studies using the DMQ-R have focused on students and young adults. In recent years, the DMQ-R has been applied also in studies in the general population [7, 8]. Although the questionnaire was developed for non-clinical samples, it has recently been used in studies of persons with alcohol use problems [3, 9]. Further, in a recent study our group could confirm the four-factor structure of the Swedish version of the DMQ-R in a group of adults seeking outpatient psychiatric care [10].

To evaluate the performance of a questionnaire, direct study of the question-and answer process is recommended [11, 12]. Still, only a small number of studies have investigated how respondents in health care settings interpret questions and statements and what their thoughts are when doing so [13–16]. Results from those studies indicate that, although well validated with standard psychometric methods, questionnaires are not always perceived as intended: responders may interpret questions in unexpected ways and have difficulties understanding them [13]. To our knowledge, no such studies have been performed in a psychiatric setting.

The DMQ-R has not been investigated with qualitative methods. To extend the knowledge about the psychometric properties, the usability and utility of the DMQ-R in different settings, a project called “Motives for drinking” was initiated (for previous reports, see [3, 10]). This study is a part of that project, aiming to further investigate the benefits and shortcomings of the questionnaire by studying how respondents perceive and interpret the questionnaire. The specific aim of this study was to provide qualitative knowledge of acceptability, accuracy and usability of the DMQ-R among persons receiving outpatient psychiatric care by studying how responders perceive and interpret the questionnaire.

## Method

### Setting and participants

Data were collected among patients visiting a psychiatric outpatient clinic for mood and anxiety disorders at a university hospital in Sweden. The hospital’s procedure includes referring those patients who have a substance use disorder or psychosis as a main diagnosis to separate specialist clinics and those patients did not take part in this study. In the eligible group of patients with mood and anxiety disorders, co-morbidity with personality disorders or ADHD/autism spectrum disorders may occur, but no patient was diagnosed with any substance use disorder. The only criterion for inclusion was having consumed alcohol during the last 12 months. All staff at the outpatient unit were asked to inform and invite their patients to participate in the study. No compensation was offered. The project was approved by the Ethical Review Board of Uppsala University (Reg. No. 2015/434).

### The Drinking Motives Questionnaire-Revised (DMQ-R)

The Drinking Motives Questionnaire-Revised (DMQ-R) [1] measures four types of drinking motives: enhancement, coping, conformity and social. Drinking to increase positive mood reflects an internal, positive-reinforcement *Enhancement* motive for alcohol consumption (e.g. drinking to feel high). Drinking to reduce negative affect reflects an internal, negative-reinforcement *Coping* motive (e.g. drinking to forget problems). Alcohol consumption to avoid negative social consequences reflects an external, negative-reinforcement *Conformity* motive (e.g. drinking to fit in with others). Drinking in order to obtain social rewards reflects an external, positive-reinforcement *Social* motive (e.g. drinking to celebrate with friends).

The DMQ-R consists of 20 items, five per dimension. Participants rated the frequency of drinking for each item on an ordinal scale with six response categories (1 = never, 2 = almost never, 3 = some of the time, 4 = about half of the time, 5 = most of the time, 6 = almost always). To avoid space-demanding iterations, all items were not fully written out. The questionnaire and its graphic design is presented in Additional file 1.

### Data collection

The think-aloud method was used to collect data. This method, also known as protocol analysis, was introduced in the field of psychology in the 1980s as a way to understand elementary cognitive processes as they unfold over time [11, 17]. To provide information about their thought processes, respondents are asked to “think aloud” while completing a task [17]. The researcher assumes a non-active position in the room and only interferes to remind respondents to keep thinking aloud. The think-aloud method has been used to investigate self-report questionnaires and is also recommended for cognitive pre-testing of survey questions [11–16, 18]. Findings can be used to refine measures or highlight areas for consideration when applying them [19].

Participants in this study were offered oral and written information by their ordinary caregiver and those who chose to participate were scheduled for a session with one of the researchers MW or CN. The information pointed out that the study focused on the thoughts while answering a questionnaire rather than on the actual answers. At the session, participants were informed again and signed a consent form. They were instructed to think aloud: to say whatever came into their mind while responding to the questionnaire. They were informed that they could be reminded to keep talking, and that questions about items would not be answered by the researcher. If participants remained silent for 5–7 s when completing the DMQ-R, they were asked what they were thinking about. In case verbalized thoughts were unclear, participants were asked to explain more explicitly. At the end of the session, participants were asked what they thought of filling out the questionnaire and what its pros and cons were. They were also asked if they thought something was missing. All sessions were recorded with MP3-players and transcribed verbatim.

### Data analysis

To analyse the think-aloud data, the researchers conducted Qualitative Content Analysis using an inductive approach [20]. Themes were identified and analysed inductively, i.e. themes were formed from data only and not from predetermined areas of interest.

The transcripts were read several times by authors CN and MW separately. Meaning units—words and sentences of interest for the aims of the study—were coded. Following a joint discussion, codes were sorted into themes. The material was re-read by both authors CN and MW and themes were used to sort the codes. The analysis continued until all themes were deemed to be clearly defined and distinct from one another. All authors discussed the coding of data until consensus was achieved and themes were perceived as describing the content concisely. In addition to being researchers, all authors have extensive experience in the field of psychiatric care and/or substance use treatment.

## Results

Of the 16 individuals included, 12 were women and four were men None of them was diagnosed with an alcohol use disorder but all had consumed alcohol during the past 12 months. All those approached except for one patient consented to take part. Ages ranged from 26 to 67; mean age (SD) was 45.2 (10.9), median was 46.5 years. All participants were diagnosed with a mood or anxiety disorder (ICD-10 F30-48 [21]) as a main diagnosis. The interview sessions were between 3:43 and 29:33 min long with a median length of 10:39 min. All interviews were included in the analysis.

The results are presented under the three identified themes with verbatim quotes to illustrate the findings. The themes are *Interpretation of the questionnaire, Experiences rather than motives* and *Self*-*reflection.*

### Theme: interpretation of the questionnaire

In all, 88 verbalizations concerned the interpretation of the questionnaire: the graphic design (43 comments), the formulation of questions (31 comments) and the lack of suitable response options (14 comments). The graphic design was initially confusing to some participants, a subject that generated 16 verbalizations.

How often do you drink…here, I am probably not supposed to fill in on the dotted lines, but in the box… [reads the response options]. So this is some kind of headline to this section… (#5, male 49 yrs)

The fact that questions could be posed repeatedly in similar forms produced 17 comments.

Although participants generally understood the questions, some words and terms were perceived as equivocal. In all, 31 verbalizations concerned interpretations of the questions. The concept “how often” was sometimes difficult to quantify. It was associated with consumption level and not with comparisons to other times the person had consumed alcohol. Ten verbalizations were noted on this subject.

‘Some of the times’, that’s very relative. To me, ‘soon’ is within ten minutes. I have a friend, he thinks ‘soon’ is within an hour. It’s just about the same with ‘some of the times’. (#9, female 36 yrs)

Another concept that was perceived equivocally was getting high (Item no. 9: “How often do you drink to get high?”) One participant interpreted “to get high” as “to be intoxicated” or “really drunk”. Another interpreted the wording as “slightly affected by alcohol”. Still another participant contemplated:

…it depends on what you mean by ‘high’. To get high, that’s to be sort of upbeat to me. But I mostly want to calm down, so no… (#4, female 38 yrs)

The participants sometimes had difficulty relating to the situation described in the question.

…because it makes social gatherings more fun. It depends on the context…if I go out after work with some colleagues, maybe we’ll drink wine together. But other times it’s a family gathering and I may as well drink cola…What should I put here? This was really difficult. (#13, female 49 yrs)

Participants were eager to fulfil their task, but in all 14 verbalizations indicated difficulty finding a suitable response option. Instead, they chose a rough estimate.

…to celebrate…well, no…I put ‘almost never’, it is… it is mostly because I want to move on from the question and I don’t want to leave the questionnaire incomplete. (#10, female 53 yrs)

### Theme: experiences rather than motives

When participants reflected on the questions it was common to refer to practical experiences, situations the person had been in. Those experiences – rather than the motive for drinking—seemed to guide the response. This theme gathered 29 verbalizations.

…because it gives you a pleasant feeling. Yes, to me it does, it makes me calmer. Because it makes you feel more self-confident…yes, that is common. (#4, female 38 yrs)

…because it gives you a pleasant feeling. If you drink moderately, yes. But if you drink too much, absolutely not. I get really sick from it. (#2, female 34 yrs)

### Theme: self-reflection

The questions led some of the participants to reflect on their alcohol use and motives. This was spontaneously commented on six times.

You really start reflecting on your drinking. Something happens to you when you’re asked these questions. […] You really confront yourself with these questions, and I think that might be a good thing. It could be a kind of an opening to your understanding that this isn’t good, maybe. (#13, female 49 yrs)

I think these questions are…they make it difficult to lie. It’s easier to lie if you are asked how much, how many beers did you drink. (#8, male 35 yrs)

### Additional observations

Participants who were insecure about how to reply often chose the alternative “Some of the times”.

“To get high. [long pause]. Hm. I don’t really know. But maybe we can say “Some of the times”. (#2, female 34 yrs)

The participants that ruled out conformity motives did so particularly decisively.

Because your friends pressure you to drink…Never! I drink when I decide to. And if I don’t feel like drinking when somebody offers me a drink, I just say no thanks. (#11, male 44 yrs)

Some participants commented that they found the questionnaire non-confrontational and exhaustive.

I think…they were good questions, they were neutral. Not moralistic… they don’t ask how often you drink, and so on. And they were comprehensive, I can say. They approach the question of why you drink from all kinds of angles. (#15, female 55 yrs)

Others pointed out that it covered important areas that no one had asked them about before.

Five responders commented that an important drinking motive was that alcohol tastes good and goes well with food. One person felt a response option was missing for questions about which she was uncertain.

## Discussion

This is the first study to report qualitative data from thought processes of persons responding to the DMQ-R. In our study, we were able to find out more about interpretations and reasoning among the psychiatric outpatients. Information collected using the think-aloud method would have been difficult to collect with fixed response options or by asking participants retrospective questions.

The acceptability of the questionnaire was generally high; it was well understood by the participants. However, there were many comments concerning similarities among the questions and the iterations seemed to create some vexation and insecurity. Thus, a shorter version may be to prefer. In a previous study with patients seeking psychiatric care, we found psychometric properties of the short form DMQ-R SF to be valid and even with a slightly better model fit than the DMQ-R [20]. The results of this study add to the preference for the use of the short form of DMQ-R.

A large number of verbalizations pertained to the questionnaire itself: its graphic design, the formulation of questions and the lack of suitable response options. Some respondents perceived the graphic design used in our study as confusing. For simplification, the questions were not fully written out. One patient broke off the interview after 4:30 min, as he felt his symptoms produced an uncomfortably stressful situation. Two other participants mentioned their ADHD symptoms as a possible explanation for their difficulties understanding the questionnaire. When addressing patients in psychiatric care there may be a certain need for explicitness, as their psychiatric condition may create cognitive disturbances.

The verbalizations concerning difficulties finding an apt response option indicate that some tentativeness is needed when interpreting the results of the questionnaire. The option “Some of the time” was often chosen when responders were insecure about how to reply. The responses “Some of the time” may thus represent low accuracy.

Before giving a response, many participants considered the effects alcohol has on them rather than their motives for drinking. It was clear that they were referring to their experiences and the consequences of drinking. In cognitive psychological theory, the frequently cited “Question-and answer model” (e.g. [11].) suggests that respondents must complete four actions to be able to answer a question: they must comprehend the question, retrieve the necessary information from long-term memory, make a judgement about the information needed to answer the question, and respond to the question. Thus, the thought process that leads to an answer include considering previous experiences and that became evident in the think-aloud interviews. Therefore, we do not deem this reasoning to reduce the validity of the questionnaire.

It is noteworthy that respondents spontaneously commented that the questionnaire made them start reflecting on their alcohol use. Although such verbalizations were quite rare during the interviews, considering the nature of those comments, it is likely that the questions on the DMQ-R may launch a self-reflection process. Because alcohol use even at moderate levels may impact the course of a psychiatric disorder and the treatment effects [22, 23], such processes may help prevent possible exacerbations due to alcohol. Furthermore, participants in this study perceived the questionnaire as non-confrontational and felt that it covered important areas that no one had asked them about before.

The comments concerning the graphic design generated the major share of comments. This serves as a reminder that a clear and comprehensible design is of importance, in particular in the psychiatric setting. However, design issues are easily resolved. It is more problematic that respondents differed in the interpretation of questions and situations described. Previous psychometric tests of DMQ-R have pointed out variations in item factor loadings in groups of adults as well as adolescents [8, 24, 25]. For example, in some studies item no. 9 (“…to get high”) yielded a lower factor loading than other enhancement items among adults. Item no. 17 (…“because it’s fun”) produced a low factor loading in the enhancement motive among European students [26]. These inconsistencies may illustrate the fact that questionnaires do not pinpoint a precise measure of people’s views and feelings. The result of a self-report questionnaire as reported in numbers and decimals may lead the interpreter into thinking that it is an exact measure of the responder’s thoughts and beliefs. In this as well as in previous studies, findings suggest that questionnaires can be understood in unforeseen ways and that responses may be given with less accuracy than expected. We recommend caregivers as well as researchers to take this into consideration when evaluating results obtained by self-report questionnaires. Keeping this in mind, results from this as well as previous studies still suggest that the DMQ-R is of value for research, prevention and treatment purposes in patients with mood and anxiety disorders.

There are some issues to consider with respect to strengths and measures used to strengthen the weaknesses of the present study. The main strength of this study is that it investigates the questionnaire from the respondent’s perspective. It was conducted in a clinical setting and used a cognitive method, previously not used with DMQ-R, to examine how people understand and interpret the questionnaire.

One potential weakness is the interview setting with a researcher present in the room that may have created bias; given that the subject is alcohol, social desirability may have played a role. Still, because of the non-confrontational nature of the questions we believe that risk was small. Further, it is possible that the think-aloud method may have increased the responders’ critical attitude to questions in a way that differs from a normal response situation.

As with all research with qualitative analysis, there must be measures to strengthen the neutrality of the coder, which otherwise may affect the interpretations. Nevertheless, interpretation involves a balancing act between adding a perspective to the phenomenon under study and not assigning meaning to the text that is not there. The co-authors reduced that risk via a sound data analysis process with alternative coding and discussions of the data. Further, although the coders were experienced in psychiatric care and/or substance use treatment they were not members of the staff and had not met the participants before, which minimized the influence of pre-existing assumptions.

This type of study design gives a good understanding of perceptions in a group of individuals, but it is not possible to generalize the results from the study in a quantitative manner. Instead, description of context, process of analysis and appropriate quotations can inform and enhance readers’ understandings of how the findings can be transferred to other settings or groups.

The number of participants was relatively small, however qualitative research is often based on small sample sizes where understanding of experiences and perceptions is the subject. Nevertheless, a larger sample could have made it possible to include participants of different ages, different diagnoses and different experiences of alcohol.

## Conclusions

The results suggest a support for the use of DMQ-R in outpatients in psychiatric care for research, prevention and treatment purposes. Still, when interpreting the DMQ-R, a certain insecurity of the exactness of answers should be considered. The graphic design should be particularly clear in this group of patients.

## Additional file

**Additional file 1.** The Drinking Motives Questionnaire in the version used in the study. English translation supplied.
