# Supplementary material for: How do people with mood and anxiety disorders perceive and interpret the Drinking Motives Questionnaire? A think-aloud study in a clinical setting
Source: Addict Sci Clin Pract. 2018 Mar 14;13:7. doi: 10.1186/s13722-018-0109-1 (PMC5851067; doi:10.1186/s13722-018-0109-1)
Supplement: Supplementary file 1 — Additional file 1. The Drinking Motives Questionnaire in the version used in the study. English translation supplied. [file 13722_2018_109_MOESM1_ESM.pdf]

DMQ – Drinking Motives Questionnaire
Motiv för att dricka alkohol

Hur gammal är du? ………..år

Man ☐ Kvinna ☐

Här följer några skäl som människor uppgett till att de dricker alkohol. Använd svarsalternativen när du anger dina skäl. Inget svar är rätt eller fel.

Hur ofta dricker du……. (How often do you drink...)	Aldrig	Nästan aldrig	Ibland	Ungefär hälften av de gånger jag dricker	De flesta gånger jag dricker	Nästan varje gång jag dricker
1. för att du tycker om känslan det ger? (because you like the feeling?)	☐	☐	☐	☐	☐	☐
2. för att glömma dina bekymmer? (to forget your worries?)	☐	☐	☐	☐	☐	☐
3. för att du känner dig pressad av andra att göra det? (because your friends pressure you to drink?	☐	☐	☐	☐	☐	☐
4. för att det hjälper dig att ha kul på en fest? (because it helps you enjoy a party?	☐	☐	☐	☐	☐	☐
5. för att det gör dig upprymd? (because it's exciting?)	☐	☐	☐	☐	☐	☐
6. för att det hjälper dig när du känner dig nedstämd eller orolig? (because it helps you when you feel depressed or nervous?)	☐	☐	☐	☐	☐	☐
7. för att slippa kommentarer från andra? (so that others won't kid you about not drinking?)	☐	☐	☐	☐	☐	☐
8. för att vara social? (to be social?)	☐	☐	☐	☐	☐	☐
9. för att få ett rus? (to get high?)	☐	☐	☐	☐	☐	☐
10. för att muntra upp dig när du är på dåligt humör? (to cheer up when you're in a bad mood?)	☐	☐	☐	☐	☐	☐

Hur ofta dricker du…….	Aldrig	Nästan aldrig	Ibland	Ungefär hälften av de gånger jag dricker	De flesta gånger jag dricker	Nästan varje gång jag dricker
11. för att passa in? (to fit in with a group you like?)	☐	☐	☐	☐	☐	☐
12. för att det blir roligare när du träffar andra? (because it makes social gatherings more fun?)	☐	☐	☐	☐	☐	☐
13. för att det ger en behaglig känsla? (because it gives you a pleasant feeling?)	☐	☐	☐	☐	☐	☐
14. för att få bättre självförtroende eller känna dig mer säker på dig själv? (because you feel more self-confident or sure of yourself?)	☐	☐	☐	☐	☐	☐
15. för att bli accepterad? (to be liked?)	☐	☐	☐	☐	☐	☐
16. för att det gör fester roligare? (because it improves parties and celebrations?)	☐	☐	☐	☐	☐	☐
17. för att det är roligt? (because it's fun?)	☐	☐	☐	☐	☐	☐
18. för att glömma dina problem? (to forget about your problems?)	☐	☐	☐	☐	☐	☐
19. för att inte känna dig utanför? (so you won't feel left out?)	☐	☐	☐	☐	☐	☐
20. för att fira tillsammans med vänner? (to celebrate a special occasion with friends?)	☐	☐	☐	☐	☐	☐
